# Prevalence of Diabetic Macular Edema Using Optical Coherence Tomography in Type 2 Diabetics With Nephropathy in Comparison With Type 2 Diabetics Without Nephropathy

**DOI:** 10.7759/cureus.70703

**Published:** 2024-10-02

**Authors:** Vinisha Kumaresan, Abinaya Ramakrishnan, Nithya R, Divya N

**Affiliations:** 1 Ophthalmology, Saveetha Medical College and Hospitals, Saveetha Institute of Medical and Technical Sciences, Saveetha University, Chennai, IND

**Keywords:** diabetic macular edema, diabetic nephropathy (dn), optical coherence tomography, prevalence, type 2 diabetes

## Abstract

Introduction: The primary vision-threatening complication in patients with diabetic retinopathy (DR) is diabetic macular edema (DME). Diabetic nephropathy (DN) often has DR that threatens their vision and has a risk of developing DME. Hence, the presence of DN could be the only risk factor for developing DME without the presence of other factors. Hence, this study proposes to predict the prevalence of DME using optical coherence tomography (OCT) in type 2 diabetics with nephropathy in comparison with diabetics without nephropathy.

Methods: This is a cross-sectional study done on patients visiting the Ophthalmology Department at Saveetha Medical College and Hospital, Chennai, for one year. A total of 120 patients were included in the study, with 60 diabetic patients without nephropathy in Group 1 and 60 diabetic patients with nephropathy in Group 2. A detailed history was recorded. Their recent blood reports of fasting blood sugar, postprandial blood sugar, hemoglobin A1c (HbA1c), renal function test, and urine protein/creatinine ratio were all noted. Chronic kidney disease (CKD) staging in nephropathy patients as done by the nephrologist was also documented. The best corrected visual acuity (BCVA) was assessed for distant and near vision. A slit lamp examination and dilated fundus examination were done. Fundus findings were independently graded according to the Early Treatment Diabetic Retinopathy Study (ETDRS) classification. OCT was done in all patients to confirm DME.

Results: In our study. males and females were 60 each in number. The majority of patients (32.5%) were >70 years old. A small percentage of patients (1.7%) in Group 1 had DM of >10 years, and 41.7% of the patients in Group 2 had DM of >10 years. Meanwhile, 73.3% of the patients in Group 1 had an HbA1c level between 6% and 7.5%, and 83.3% of the patients in Group 2 had an HbA1c level >7.5%. The percentage of patients with DME in Group 1 was 1.7%, and that in Group 2 was 21.7%. The majority of patients who had BCVA of 6/6 were in Group 1. The majority of patients with DME (46.4%) had stage V nephropathy.

Conclusion: The coexistence of nephropathy in diabetic patients increases the incidence of DME. With long-standing diabetes and uncontrolled blood sugar levels being risk factors for developing DN and subsequently DME, such patients are to be screened regularly and advised on adequate glycemic control for achieving good visual prognosis and limit retinopathy progression. Moreover, the severity of DN should be kept in mind by nephrologists, and patients are to be promptly referred to the ophthalmology department for complete evaluation.

## Introduction

Diabetic microvascular consequences include diabetic nephropathy (DN) and diabetic retinopathy (DR). DR usually affects the population of working age with the primary vision-threatening complication in patients with DR being diabetic macular edema (DME) [1]. Optical coherence tomography (OCT) is an objective tool that can be used for the quantitative measurement of macular edema [2].

DME develops as a result of vascular hyperpermeability and excessive vascular leakage of fluids, proteins, or lipids in the macular region, which is brought on by microstructure disarray and microcirculation dysfunction [3,4]. The main cause of chronic kidney disease is DN, which is characterized by persistent albuminuria, progressive reduction of filtration rate, and elevated blood pressure. Albumin in the urine indicates glomerular damage, as well as systemic endothelial abnormalities and vasculopathy [5,6].

It has been observed that diabetic patients who have proteinuria or are receiving dialysis often have DR that threatens their vision and has a risk of developing DME [7]. According to Hsieh YT et al., microalbuminuria remission protects against proliferative diabetic retinopathy (PDR) and DME development, while microalbuminuria progression could lead to PDR development [8]. Therefore, the presence of DN could be the only risk factor for developing DME without the presence of other factors.

As the number of patients diagnosed with diabetes mellitus is increasing in the community, the number of patients developing vision-threatening DME is also increasing [2]. With nephropathy being a proven risk factor for DR, this study is proposed to predict the prevalence of DME in type 2 diabetics with nephropathy in comparison with diabetics without nephropathy using OCT.

## Materials and methods

This cross-sectional study was conducted from January 2021 to December 2021 for a period of one year in patients visiting the ophthalmology department and nephrology department at Saveetha Medical College and Hospital, Chennai.

Inclusion criteria

The study included type 2 diabetic patients with normal renal parameters by a consultant physician and type 2 diabetic patients clinically diagnosed with DN by a nephrologist. Patients who agreed to be part of the study of either sex with age of more than 40 years were included.

Exclusion criteria

Patients who underwent ocular surgery in the past three months, with media haze that made it difficult to get OCT images, those with hypertension and other systemic conditions causing retinopathy, and patients with other macular pathologies were excluded.

The Institutional Ethics Committee of Saveetha Medical College and Hospital issued approval SMC/IEC/2020/12/037. The sample size was calculated using the CDC Epi info application. A total of 120 patients fulfilling the inclusion criteria and willing to be part of the study were included after informed consent. Sixty diabetic patients with normal renal parameters were added to Group 1, and 60 diabetic patients with deranged renal parameters and clinically diagnosed as DN by the nephrologists were added to Group 2. The nephrologists graded the renal function based on the estimated glomerular filtration rate (eGFR), which was defined as normal (stage I) (eGFR > 90 mL/min/1.73 m^2^), mildly impaired (stage II) (eGFR 60-89 mL/min/1.73 m^2^), or chronic kidney disease (stage IIIa, IIIb, IV, V) (eGFR < 60 mL/min/1.73 m^2^).

A detailed history was recorded, which included the duration of diabetes, history of any other comorbidities including hypertension and coronary artery disease, and history of ocular surgery. Their recent blood reports of fasting blood sugar, post-prandial blood sugar, hemoglobin A1c, renal function test, and urine protein/creatinine ratio were all noted. Chronic kidney disease (CKD) staging in nephropathy patients as documented by the nephrologist was also documented. Vitals (blood pressure, pulse, respiratory rate) were assessed. Best corrected visual acuity (BCVA) was assessed for distant and near vision using Snellen's chart and near vision chart. Detailed slit lamp examination of the anterior segment was done, the patients were dilated, and the fundus findings were independently graded according to the Early Treatment Diabetic Retinopathy Study (ETDRS) classification. OCT was done in all patients to confirm DME.

Analysis of collected data was done using IBM SPSS Statistics for Windows, Version 25.0 (released 2016, IBM Corp., Armonk, NY). Descriptive analysis was used to find frequency and percentages. The chi-square test and Fischer exact test were used in the present study. A p-value <0.05 was considered statistically significant.

## Results

Association of the demographic and diabetic profiles between the two groups

Among the patients in Group 1, the majority (36.7%) were aged 51-60 years, whereas in Group 2, the majority (38.3%) were aged 61 -70 years. Gender distribution was similar between the two groups, with 28 males and 32 females in Group 1 and 32 males and 28 females in Group 2. Age and gender distribution did not have any statistical significance between the two groups.

Notably, the duration of diabetes varied significantly between the two groups, with only 1.7 % of the patients in Group 1 having diabetes for >10 years while 41.7% of the patients in Group 2 had diabetes for >10 years. In addition, most of the patients (73.3%) in Group 1 had HbA1c levels between 6% and 7.5%, while most in Group 2 (83.3%) had HbA1c levels >7.5%, as can be seen in Table 1.

**Table 1:** Association of the demographic and diabetic profiles between the two groups by the chi-square test *p < 0.05, ***p < 0. 001 - statistically significant, ns- not significant

Parameter	Group 1 N (%) N = 60	Group 2 N (%) N = 60	Total N (%) N = 120	X^2^ value	p-value
Age distribution					
40 -50	06 (10)	02 (3.3)	08 (06.7)	6.198	0.101 (ns)
51 -60	22 (36.7)	14 (23.3)	36 (30)		
61 -70	14 (23.3)	23 (38.3)	37 (30.8)		
>70	10 (30)	21 (35.0)	39 (32.5)		
Gender					
Male	28 (46.7)	32 (53.3)	60 (50)	0.533	0.465 (ns)
Female	32 (53.3)	28 (46.7)	60 (50)		
Duration of Diabetes Mellitus					
>5	06 (10.0)	0	06 (05.0)	35.559	0.000***
5 -10	53 (88.3)	35 (58.3)	88 (73.3)		
>10	01 (1.7)	25 (41.7)	26 (21.7)		
HbA1c					
6-7.5	44 (73.3)	10 (16.7)	54 (45)	38.923	0.000***
>7.5	16 (26.7)	50 (83.3)	66 (55)		

Comparison of the demographic and diabetic profiles between the two groups

The mean age of our studied population was 64.68 ± 9.18 years with mean age in Group 1 and Group 2 being 63.7 ± 9.93 years and 65.65 ± 8.44 years, respectively, and it was not statistically significant. The patients in Group 2 as compared to patients in Group 1 had a significantly longer duration of diabetes (11.2 ± 3.65 vs. 7.6 ± 1.70 years) and poorer glycemic control, with higher fasting blood sugar (157.60 ± 23.94 vs. 145.03 ± 22.50 mg/dL), postprandial blood sugar (289.02 ± 51.42 vs. 235.35 ± 43.37 mg/dL), and glycated hemoglobin (8.84 ± 1.25 vs. 7.34 ± 0.70%) levels, as can be seen in Table 2.

**Table 2:** Comparison of the demographic and diabetic profiles between the two groups by Student's t-test *p < 0.05, ***p < 0. 001 - statistically significant, ns - not significant

Parameter	Group 1 Mean ± SD N =60	Group 2 Mean ± SD N =60	t – value	p - value
Age	63.7 ± 9.93 years	65.65 ± 8.44 years	-1.128	0.261 (ns)
Duration of diabetes mellitus	7.6 ± 1.70 years	11.2 ± 3.65 years	-6.850	0.000***
Fasting blood sugar	145.03 ± 22.50 mg/dL	157.60 ± 23.94 mg/dL	-2.962	0.004***
Post-prandial blood sugar	235.35 ± 43.37 mg/dL	289.02 ± 51.42 mg/dL	6.179	0.000***
HbA1c	7.34 ± 0.706 %	8.84 ± 1.25 %	-8.100	0.000***

Association of DME between the two groups

In the studied groups, a significant difference in the prevalence of DME was noted among the two groups. More number of patients (21.7%) in Group 2 presented with DME, while only 1.7% patients in Group 1 was noted to have DME, as can be seen in Table 3.

**Table 3:** Association of diabetic macular edema between the two groups by chi-square test *p < 0.05, ***p < 0. 001 - statistically significant, ns - not significant

Diabetic macular edema	Group 1 N (%) N = 60	Group 2 N (%) N = 60	Total N (%) N = 120	X^2^ value	p-value
Present	01 (1.7)	13 (21.7)	14 (11.7)	11.644	0.000***
Absent	59 (98.3)	47 (78.3)	106 (88.3)		

In Group 1, 53 patients (88.3%) had the best corrected distant vision of 6/6, whereas in Group 2, only 16 patients (26.6%) had the best corrected distant vision of 6/6 in atleast one of their eyes.

Association of stage of DN with DME

According to their eGFR, DN was categorized into various stages including stage IIIa, stage IIIb, stage IV, and stage V. A significant association between DME and stages of DN was noted, with 46.4% of DME present in stage V, 38.4% in stage IV, and 7.6% each in stages IIIa and IIIb, contrasting with its absence in 40.4% in stage IV, 29.7% in stage IIIa, 19.3% in stage IIIb, and 10.6% in stage V, as can be seen in Table 4.

**Table 4:** Association of the stage of diabetic nephropathy with diabetic macular edema by using the chi-square test *p < 0.05, ***p < 0. 001 - statistically significant, ns - not significant

Stages of diabetic retinopathy	Diabetic macular edema			X^2^ value	p - value
	Present N (%) N = 13	Absent N (%) N = 47	Total N (%) N = 60		
Stage IIIa	01 (7.6)	14 (29.7)	15 (25)	9.806	0.020***
Stage IIIb	01 (7.6)	09 (19.3)	10 (16.6)		
Stage IV	05 (38.4)	19 (40.4)	24 (40.0)		
Stage V	06 (46.4)	05 (10.6)	11 (18.3)		

## Discussion

In our study, the gender distribution was equal with 60 females and 60 males with no specific gender preponderance. Meanwhile, a similar study by Hsieh YT et al. showed female preponderance and a study by Koo NK et al. showed male preponderance [8,9].

The mean age of our studied population was 64.68 ± 9.18 years, which is comparable to a study by Hsieh YT et al. where the mean age of the studied population was 63.4 ± 11.9 while a study by Suzuki Y et al. revealed the mean age to be 70.1 ± 10.1 years [8,10]. However, our study did not show any statistical significance among the two groups with respect to the age group.

In our study, we have observed that only 1.7% of the patients in Group 1 had DM for >10 years, whereas 41.7 % of the patients in Group 2 had DM for >10 years with the mean duration in Group 1 and Group 2 being 7.6 ± 1.70 years and 11.2 ± 3.65 years, respectively. This is comparable to previous studies that showed that patients having diabetes for a longer period of time have an increased chance of developing nephropathy [11,12]. Moreover, the chances of end-stage renal disease in patients with DN increase with the longer duration of diabetes, and the mortality risk also increases [13,14].

Higher HbA1c levels are linked to an increased risk of nephropathy in patients with type 2 DM [15,16]. Several observational studies found that diabetic patients who attained adequate glycemic control had a significant reduction in the occurrence of DN [17]. Likewise, in our study, also we noticed that the majority of the patients (83.3%) with DN had HbA1c levels >7.5 g/dl while the majority of patients (73.3%) without DN had HbA1c levels between 6 and 7.5 g/dl.

It was noted that only one patient (1.7%) in Group 1 had DME, whereas 13 patients (21.7%) in Group 2 had DME. Although the majority of the patients in Group 1 and Group 2 did not have DME, patients with nephropathy developed DME more as compared to patients without nephropathy. This is similar to a study by Acan D et al., which stated that DN is a significant factor in DME development [17].

The best visual acuity of 6/6 was mainly noted in patients without nephropathy (88.3%) and only a few patients with nephropathy (26.6%) had the best visual acuity of 6/6. Poor visual acuity is seen more commonly in patients with nephropathy. This could be attributed to the increased prevalence of DME in nephropathy patients as compared to patients without nephropathy.

Moreover, it could be observed that in our study DME prevalence was higher in patients with a severe grade of DN as the majority of patients with DME (46.4%) had stage V nephropathy. This is in accordance with Acan D et al. where it was stated that the severity of DN is also a significant factor for DME prevalence in addition to other risk factors [17]. Hence, it is crucial to note that both DR and DN are risk factors for each other as the presence of one leads to the progression of the other [18-20].

Since the study included a small sample size and it was a cross-sectional, single-center study, the results cannot be generalized. Moreover, clinically diagnosed nephropathy cases were included in our study, but the results would be more reliable when the study was done in biopsy-proven nephropathy cases.

## Conclusions

This study highlights that diabetic patients who have coexisting nephropathy have increased chances of developing DME. Long-standing diabetes, especially more than 10 years, and uncontrolled blood sugar levels are risk factors for developing DN, which will ultimately lead to the development of DME. With DME being one of the important causes of vision loss in diabetic patients, such patients on identification are to be screened regularly and advised on adequate glycemic control for achieving a good visual prognosis. This also helps in limiting retinopathy progression as DR and DN are risk factors for each other. In addition, nephrologists should keep in mind to promptly refer all diabetic patients with nephropathy to the ophthalmology department for complete evaluation. Patients with severe grades of DN are to be explained about the poor visual prognosis while the less severe cases of DN are to be managed timely and appropriately to preserve their vision at the earliest.
